# Object-centered family interactions for young autistic children: a diary study

**DOI:** 10.1038/s41598-024-55177-4

**Published:** 2024-03-05

**Authors:** Yuqi Hu, Xu Sun, Cheng Yao, Shijian Luo, Bingjian Liu, Mengru Xue, Hui Lyu

**Affiliations:** 1 Faculty of Science and Engineering, University of Nottingham Ningbo China, Ningbo, 315100 China; 2 Ningbo Innovation Center, Zhejiang University, Ningbo, 315100 China; 3 Zhejiang University, Hangzhou, 310058 China; 4 Nottingham Ningbo China Beacons of Excellence Research and Innovation Institute, Ningbo, 315101 China

**Keywords:** Health care, Quality of life

## Abstract

Autistic Children often struggle with social interaction and communication, studies have found that many of them prefer to interact with objects than people. However, there is a lack of research exploring the specific characteristics and factors involved in interactions within families with autistic children where objects are the center of the interaction. This paper describes the process and findings of a diary study exploring how young autistic children interact with their families through objects in natural scenarios. A one-week diary study was conducted with six families with young autistic children. Diary videos were recorded onsite and coded later according to a social interaction behavior scheme with corresponding diary entries. Qualitative data analysis was conducted to reveal possible patterns. Results revealed ongoing difficulties in establishing and maintaining family interaction and identified influential factors of object-centered family interaction. The most prevalent pattern observed was parents taking the lead in interactions, followed by the child's confirmation response. Remarkably, daily necessities emerged as potential physical mediums for enhancing family interactions, opening avenues for exploring tangible designs in human–computer interaction. These findings offer valuable implications for future research and the development of innovative designs that promote enriching interactions for autistic children and their families.

## Introduction

Autism spectrum disorder (ASD) is highly prevalent worldwide. It is a set of heterogeneous neurodevelopmental disorders characterized by alterations in social interaction and communication, as well as patterns of restrictive and repetitive behaviors (RRBs)^1^. The etiology and pathogenesis of ASD remain unknown, making it difficult to develop targeted therapies for patients^2^. As such, it is essential to prioritize the understanding and management of ASD to improve the quality of life of those affected^3^.

The field of Human–Computer Interaction (HCI) has witnessed extensive research on various technologies aimed at assisting children with ASD (e.g. games, augmented reality, virtual reality, artificial intelligence-assisted)^4^. Notably, tangible interactions, wearables, and robot technologies, which are closely related to physical entities, have been investigated for their potential to improve the well-being of children with ASD^5–7^. However, much of the existing research on object-related interventions tends to focus on pretend/symbolic play or imitation, rather than natural contexts^8–10^. Furthermore, the relationship with family interactions is often not discussed in these studies^11^. Hence, this study aims to address this research gap by examining how objects are employed and how they shape family interactions in the context of supporting children with ASD. By exploring the ways in which 'objects' are applied, this research seeks to shed light on the potential of HCI designs and technology to facilitate the establishment and maintenance of higher-quality interactions among autistic children and their families. The significance of this study lies in its focus on natural contexts and the functional aspects of objects, which have been relatively underexplored in previous research. By investigating the usage of objects within real-life situations, we can gain practical insights into how HCI design and technology can effectively support and enhance interactions between children with ASD and their families.

In this paper, we present the findings of a diary study that aims to investigate interactions in families with autistic children. Our focus is on object-centered family interaction, offering first-hand insights into the potential of everyday objects as mediums for enhancing family interactions. We aim to understand the influencing factors and patterns that emerge when family members engage with the child through objects during daily activities. Through our analysis of the diary entries, we have developed a preliminary framework to organize the identified and unidentified factors that shape object-centered family interactions.

The results emphasize that while there is a high level of affirmation among families with young autistic children, there remain difficulties in establishing and sustaining effective communication. Specifically, families often initiate interactions, with no response from children. The results align with previous research on the efficacy of toys as a means of enhancing positive changes in children’s behaviors within the realm of HCI^12^. However, our study has uncovered a previously overlooked potential in utilizing everyday necessities for object-centered interaction design. This study contributes to the existing body of knowledge by highlighting the opportunities for HCI designs and technology to play a valuable role in promoting better interactions and well-being among autistic children and their families. The findings of this research have the potential to inform the development of more effective and contextually relevant interventions for individuals with ASD.

## Theoretical background

### Family involvement and interaction of young autistic children

Family involvement and interaction are crucial aspects of the lives of children with ASD, which can have positive effects. A study by Sood et al. found that the home environment can positively impact the participation of autistic children in family activities^13^. Child-centered training combined with natural feedback within the family environment can be effective^14^ and compensates for the lack of training resources. While professional training is still critical, many experts suggest that parents should play a more active role in their child's intervention and training^15,16^. A parent-mediated early intervention approach for young autistic children has yielded statistically significant positive changes in parent–child interaction patterns, surpassing the outcomes achieved through conventional or child-centered interventions or alternative methods^17^. Moreover, interventions that involve interactive participation from both parents and children have been shown to yield even better outcomes compared to interventions where parents are educated separately. These findings underscore the importance of active parental involvement and the collaborative engagement of both parents and children in achieving optimal results in interventions for children with autism^18^.

Research on autism community related with family interaction often focuses on specific dyadic interactions, such as mother–child interactions or father-child interactions. While there have been many studies on this topic, few have attempted to describe the natural interaction that occurs within families. Family units are complex social systems, composed of members who interact and relate to one another according to family system theory^19^. There are differences in the family interaction between families with and without ASD children. For example, mothers may use fewer social verbal cues and more physical contact towards their ASD children compared to their non-ASD children^20^. Parenting behavior and family interaction styles can also have different effects on the child and the family as a whole^21^. Controlling parenting styles have been associated with more externalizing problems in children, while parents who do well at following the focus of attention of their ASD children are more likely to benefit them^22,23^.

### HCI for ASD children

The application of HCI in the field of ASD has gained global attention. Although there has been an increase in the number of research studies on ASD interventions and/or designs in recent years, there is still a lack of designs or applications that cater to note the needs of individuals with ASD. In studies that focus on the relationship among multiple users and computers, many involved other ASD peers^24,25^ or therapists instead of family members. For studies that investigate interactions for families with ASD children, many were carried out in institutional classrooms or treatment rooms environment^24,26^ where It is different from how interactions might happen in home between family members, thus, the family facets in HCI for the interaction of ASD children need exploration.

Objects of various types play a significant role in the rehabilitation of children with autism. Many studies described their special interests for the physical world, that they tend to pay more attention to the environment they are in and objects around them than to people^27^, such as lighting, rotating items and mechanical switches^24,28^. Therefore, such characteristics enable the use of HCI designs, to obtain the basic rationality and a predictable good degree of acceptance in this community, for example catching one’s eyes and forming joint attention, which are important for these special kids^29^. However, while it is true that children with autism have a greater affinity for objects, they can also experience difficulties when interacting with them^30^. RRBs such as constant stroking, shaking, or gazing, these behaviors of obsessing over specific things are vivid representatives of their unhealthy relations with objects. Therefore, there is a potential for research on how objects are naturally involved in daily interactions and the reciprocal nature of such interactions between the child, other people, and the object itself^30^.

## Methods

Ethics approval for the study was obtained from the Research Ethics Committees of the University of Nottingham Ningbo China, and the study was conducted in accordance with the Declaration of Helsinki. Informed consent was obtained from legal guardians for study participation prior to the commencement of the study.

### Diary study

Diary study is a self-reporting data collection method which can be used to capture ongoing experience, such as family interaction behaviors. Compared to retrospective interviews, which are commonly used in HCI user studies, diary study could effectively reduce the time interval between the occurrence of the event and the information recording^31^, thus retaining details that might be forgotten afterwards and increasing the integrity and reliability of the data. Also, diaries could help to reduce Hawthorne effects when compared to naturalistic study methods (e.g., observations in the field)^32^. Diary study was adopted as a feasible and effective way of collecting sensitive information while maintaining the privacy and comfort of participants in this study.

We asked participants to capture short videos of various family interactions as part of the diary. Web-based forms using Wenjuan.com gathered further information about the interactions as the other part of the diary entries. Conveniently, both video recording and form submission could easily be accomplished using a smartphone, making participation accessible for all involved.

### Participants

Families who received regular intervention from two local institutions were reached through therapists’ referrals and selected through convenience sampling. A set of specific criteria were established as follows.

Inclusion criteria: (a) *The autistic child falls within the age range of 3–6*: This age range was chosen to limit the group to young children who are in similar preschool settings and/or about to enter primary school. (b) *The participating caregiver is the primary caregiver and legal guardian of the child*: The caregiver should be the family member who has the most interaction with the child. (c) *The child is attending regular schooling or intervention classes*: Children should have similar everyday routines and opportunities for parent–child interaction. (d) *Urban family*: This criterion ensures that each child does not have significantly different living environments. Exclusion criteria: (a) *Families with more than one autistic child.*

In total, eight families were recruited, but only six families met the requirements to participate due to insufficient interaction clips and diary entries. The participants' descriptions are outlined in detail in Table 1. The severity of ASD is categorized into three levels, according to the levels of support required^1^. The diagnosis provided by doctors was based on the Childhood Autism Rating Scale (CARS)^33^, while parents' ratings were influenced by their own understanding of autism spectrum disorder (ASD) and their perception of their child's condition. Interestingly, it was observed that parents generally perceived their children's condition to be more severe compared to the assessments made by doctors. Characteristics are determined by social communication difficulties and RBBs^1^ with assessments measured on a scale from 1 to 5. A score of 1 represents the mildest manifestation. Furthermore, the children's IQ scores were assessed using the Chinese version of the Wechsler Intelligence Scale for Children (C-WISC), which had been modified by Hunan Medical University^34^. This assessment was conducted to gather additional information about the cognitive functioning of the participants.

**Table 1:** Participant description.

Number	1	2	4	6	7	8
Gender	Male	Male	Male	Male	Male	Male
Age of the child (mean = 4.33, SD = 0.94)	5	4	4	4	6	3
Level of severity of ASD as rated by DOCTOR (using CARS)	Level 1	N/A	Level 1	Level 1	N/A	N/A
Level of severity of ASD as rated by PARENTS	Level 1	Level 2	Level 2	Level 1	Level 1	Level 1
Family assessment-Deficits in social communication and interaction (1–5)	2	4	3	1	2	3
Family assessment- RRBs, interests, or activities (1–5)	2	4	1	2	2	3
IQ	above 70	below 70	N/A	N/A	N/A	N/A
Functional language	Unaffected/mildly impaired	Significant impaired	Unaffected/mildly impaired	Lack/no ability	Unaffected/mildly impaired	Lack/no ability
Complication	Non	Hearing impairment	Non	N/A	N/A	Non
Relevant knowledge about ASD	Some family members have	None	None	None	None	None
Families’ appraisal of family interaction performance (1–5)	3	3	1	4	1	2
Main carer	Mother	Mother	Mother	Father	Mother	Mother
Education background of the main carer	Bachelor’s degree	Junior high school/below	Junior high school/below	Bachelor’s degree	Senior high school	Bachelor’s degree
Employment status of the main carer	Employed full-time	Housewife/househusband	Housewife/househusband	Housewife/househusband	Housewife/househusband	Employed full-time

### Procedure

Prior to the commencement of the study, participants were provided with a participant information sheet, which outlined the purpose and procedures of the research. Informed consent was obtained from all participants or their legal guardians. During an explanation session, participant families' demographic information was collected through a questionnaire, and the study procedures and tasks were thoroughly explained.

Throughout the experiment, parents were responsible for maintaining interaction diaries, documenting any instances of object-related interactions with their children within the family setting. The diaries consisted of a brief video capturing the interaction and an electronic form for supplementary information. The form collected additional background details, including the nature and purpose of the interaction, perceived functionality of the object, participants' moods, evaluations, and comments on the interaction. The videos were required to be at least 30 s in duration to allow for the identification of consecutive turns. The diary form was completed on smartphones. A minimum of one diary entry per day was necessary for valid data collection. While it was suggested to include every family member in each day's interaction diary, it was not strictly mandatory.

During the study, the primary caregiver received daily notifications without unnecessary disturbances. The study lasted one week, starting from Saturday, taking advantage of the abundant family interaction time on weekends to acquaint them with the diary recording process. After one week of recording, semi-structured interviews were conducted with the primary caregiver of each child.

### Analysis

The researchers undertook the task of translating all diary entries and interview data from Chinese to English prior to the analysis phase.

The analysis of the data primarily focused on the interaction videos, which served as the foundational and essential component. Video coding was conducted in two distinct steps, utilizing a coding system similar to the one employed by Freeman and Kasari^35^ in their study. This coding system allowed for the examination of various layers of play characteristics within the parent–child interactions in the context of autism.

Firstly, the interaction videos were transcribed into detailed and descriptive 'object-centered' behavioral accounts, capturing the actions carried out by each participant, the objects involved, the sequence of actions, and any verbal exchanges during the interactions. The transcriptions followed a turn-based interaction style, ensuring a clear depiction of the behaviors exhibited by the parent or child in each turn. Considering the common symptoms of deficits in reciprocity among individuals with ASD, even if there was no response, the turn was still recorded, with documentation of any behaviors indicative of being ignored. Table 2 shows an example clip of video transcription.

**Table 2:** An example of video transcription.

(playing with a ‘button’ toy)	
Mom04	“Put that back.” (shake the toy box)
Child04	“Oh.” (not looking at mom, throw the toy on the bed)
Mom04	“Oh, what are you going to do?”
Child04	(glance at mom and throw the toy again on the floor)
Mom04	“Put that back, put things that look the same together.”
Child04	(not looking at mom, get down to find the toy that had been thrown on the floor)

Subsequently, the transcriptions underwent coding to determine the interaction style of each participant based on their behaviors and language. We employed a moment-by-moment frequency coding approach, adapting the coding scheme utilized by Meirsschaut et al.^36^ and Bontinck et al.^20^ in their studies on mother–child and parent–child interactions in children with ASD. Additionally, we incorporated an additional category, "Ignore" to code situations where no observable reaction was evident (see complete scheme in Table 3). For each turn, the role of the parent or child was defined as exhibiting social initiative or/and social response, with specific subcategories assigned depending on the specific reactions observed.

**Table 3:** Coding scheme based on Bontinck et al.’s^20^ work.

Social initiative	Attempt to interact with someone, which can be verbal or non-verbal (e.g., pointing, showing, or seeking physical proximity combined with eye contact). It is always addressed to a person with the intention to get a response from that person	
	Declarative	Or social, to share interest in something with someone (e.g., “I’ll feed the doll”)
	Imperative	Or directive, to request something from someone (e.g., ‘‘Put that away!’’)
	Neutral	No clear declarative or imperative intention (e.g., ‘‘Ok, what’s next?’’)
Social response	Reaction to a social initiative or response and following the preceding attempt. It can be verbal and/or non-verbal and is always addressed to the other person	
	Confirming/Compliant	The response confirms the preceding initiative or response (e.g., “Yes, good idea!”)
	Non-confirming/non-compliant	The response denies the preceding initiative or response (e.g., “No, she is not hungry”)
	Neutral	The response is not clearly confirming or denying (e.g., “mmh”)
	Attempt to comply	Unsuccessful responses to comply (e.g., ‘‘I don’t know’’ as a response to question ‘‘What color is this?’’)
	Ignore	no observable response of any form

Responsiveness: the proportion of social initiatives followed by a reaction other than *Ignore.*

The diary entries served as descriptors to outline the characteristics of each interaction clip. A total of seven descriptor fields were included, as outlined in Table 4. The researchers categorized the types of objects and activities based on the collected data, while object functions were derived from previous relevant studies. Additionally, six skill and goal descriptors were included, following the guidelines of the "Autism Child Development Assessment Form (Trial)". In light of recent studies that have investigated mood as a dependent variable in interventions^37^, mood was also incorporated as one of the descriptors.

**Table 4:** Descriptors.

Descriptor field	Field options
Object type	Books and cards
	Toys
	Daily necessities and others
Object function	Reinforcer/award^38^
	Attractor^39^
	Pacifier/reliever^40^
	Tool
	Cause/the center of the interaction
Activity type	Learning and practice
	Play
	Daily activities and others
Skill and goal	Sensory perception
	Gross motor
	Fine motor
	Language and communication
	Cognition
	Social interaction
	Self-care
	Emotion and behavior
	N/A
Mood of the child/family members	Positive (e.g., exciting, happy, delighted, etc.)
	Neutral
	Negative (e.g., bored, tired, frustrated, etc.)
Value evaluation on the interaction	Positive
	Relatively positive
	Neutral
	Relatively negative
	Negative
Importance evaluation	Positive
	Negative
	N/A

To facilitate the analysis, the qualitative data analysis software Dedoose OSX 9.0.62 was employed. This software managed the coding process and facilitated the linkage between codes, descriptors, and documents throughout the analysis.

## Results

### Interaction initiatives and responses

Table 5 shows the overall interaction behavior counts of parents and children. Parents tend to be the ones initiating interaction, with the child initiating less than 20% of the interactions in this study. Both adults and children typically start or approach interactions using *Imperative* initiatives, accounting for 59.8% of children's initiatives and 76.5% of adults' initiatives. In response, *Confirming* is the most common style, with its accounting for 40% of children's responses and 53.8% of adults' responses. Therefore, the most frequent interaction style observed was *Imperative-Confirming* among all 12 combinations. This simple interaction pattern could be caused by the insufficient understanding of the pathology and related knowledge of autism among family members.

**Table 5:** Overall interaction behavior counts.

		Child		Parent		Sum
		Case count	Percentage	Case count	Percentage	Case count
Social initiative	Declarative	45	38.5	112	22.5	157
	Imperative	70	59.8	380	76.5	450
	Neutral	2	1.7	5	1.0	7
	Sum	117	19.1	497	80.9	614
Social response	Confirming/compliant	199	40.0	63	53.8	262
	Non-confirming/non-compliant	88	17.7	19	16.2	107
	Neutral	47	9.5	24	20.5	71
	Attempt to comply	13	2.6	0	0.0	13
	Ignore	150	30.2	11	9.4	161
	Sum	497	80.9	117	19.1	614
	Responsiveness		69.8		90.6	

For children, most *Imperative* initiatives involved upper limb and hand movements like pointing at objects, reaching out, pulling, or waving, with very few head movement and eye contact. Although these movements also occurred in *Declarative* initiating, children tended to use sound or verbal expression more than physical movements to attract attention or express themselves in *Declarative* initiative. For parents, there were very few *Imperative* initiatives that did not include both verbal instructions and action cues, which usually in the form of pointing, knocking objects and raising the objects in front of children’s eyes.

The overall responsiveness of child is nearly 70%, in which *Confirming* accounted for 40% of all responsive responses (any response other than *Ignore*). While this high level of response may seem promising, the prevalence of *Confirming* responses raises concerns. It was found that caregivers often provided instructions or tasks that were well within the children’s capabilities, resulting in high rates of *Confirming* responses; therefore, this may not necessarily indicate high-quality interactions. The extremely low number of *Attempt to comply* responses from children (2.6% among all responses) also partly supports the idea, it indicates that there was little room for trial or exploration, but it could also be due to children’s reluctance to make changes. It could be due to parents’ lack of knowledge and ability to scaffold interactions, so that they cannot interacting with children at or slightly above children’s current level of play. The high rate of Ignore responses at 30.2% indicates that children were often focused on their own activities and not attracted to the parent's offers (e.g., no sign of hesitation and shifting gaze). Additionally, nearly 10% of the children’s initiatives were ignored by the parents, which suggests that parents were not being responsive enough and not always following the child’s focus. Results all indicate that it is essential to improve the establishment of parent–child interactions before considering the quality of responses.

### Objects, object functions and activities

Table 6 shows the detailed case count among object type, activity types and object functions. Overall, the attitude and behaviors of parents were obviously more active and radical in interactions involving *toys*, with much less *Neutral* and *Ignore* responses. *Books and cards* were the least frequently used object type in the study. For *toys* and *daily necessities*, the excerpt numbers of them were close, but *toys* were not involved in *daily activities* and *daily necessities* were not related with *play* activities. The use of different objects in the study was consistent with our expectations. *Books and cards* were mainly used as tools for *learning and practice*, while *daily necessities* and other objects were mainly used in *daily activities* as the cause or center of the interaction. The interaction style in both situations was mainly *Imperative-Confirming*. The use of *toys* in the study was divided quite evenly between two scenarios.

**Table 6:** Descriptor × codes grid chart (object type/activity type/object function).

	All interactions initiated by both children and parents												Interactions initiated by children									
	DEclarative—attempt to comply	DEclarative—confirming	DEclarative—neutral	DEclarative—non-confirming	DEclarative—non/ignore	IMperative—attempt to comply	IMperative—confirming	IMperative—non-confirming	IMperative—neutral	IMperative—non/ignore	NEutral—neutral	NEutral—non/ignore	C Declarative—confirming	C Declarative—neutral	C Declarative—non-confirming	C Declarative—non/ignore	C Imperative—confirming	C Imperative—neutral	C Imperative—non-confirming	C Imperative—non/ignore	C Neutral—neutral	C Neutral—non/ignore
Books and cards		6	5	1	5	2	31	7	9	7		1	3	3	1	3	5					1
Books and cards × Ad		2	1				1							1								
Books and cards × Ad × Fr		2	1				1							1								
books and cards × Al		4	4	1	5	2	30	7	9	7		1	3	2	1	3	5					1
books and cards × Al × Ft		4	4	1	5	2	30	7	9	7		1	3	2	1	3	5					1
Daily necessities and others	1	32	17	7	25	6	80	40	21	35	1		9	11	2	3	15	5	4	2	1	
Daily necessities and others × Ad	1	32	17	7	24	6	74	40	21	25	1		9	11	2	3	15	5	4	2	1	
Daily necessities and others × Ad × Fr		3	2		2		1	1	1	2				2		1						
Daily necessities and others × Ad × Fa	1	3			2	2	4	1	2	3							1	1				
Daily necessities and others × Ad × Ft		5	4	5	4	2	24	25	7	9			1	2			3	1	1			
Daily necessities and others × Ad × Fc		21	11	2	16	2	45	13	11	11	1		8	7	2	2	11	3	3	2	1	
Daily necessities and others × Al					1		6			10												
Daily necessities and others × Al × Ft					1		6			10												
toys		9	10	18	21	4	104	34	8	62		5	3	2	5		28	2	7	2		
Toys × Ap		6	10	13	17	1	48	22	6	34		5	2	2			27	2	7	1		
Toys × Ap × Fa		2	5	13	7	1	46	21	5	25		5	1				26	1	7	1		
Toys × Ap × Fp		2	3		6		2	1	1	6			1	1			1	1				
Toys × Ap × Fc		2	2		4					3				1								
Toys × Al		3		5	4	3	56	12	2	28			1		5		1			1		
Toys × Al × Ft		3		5	4	3	56	12	2	28			1		5		1			1		

Empty rows were omitted.

*Al* learning and practice, *Ap* Play, *Ad* daily activities and others, *Fr* reinforcer/award, *Fa* attractor, *Fp* pacifier/reliever, *Ft* tool, *Fc* cause/the center of the interaction).

**Figure 1:**
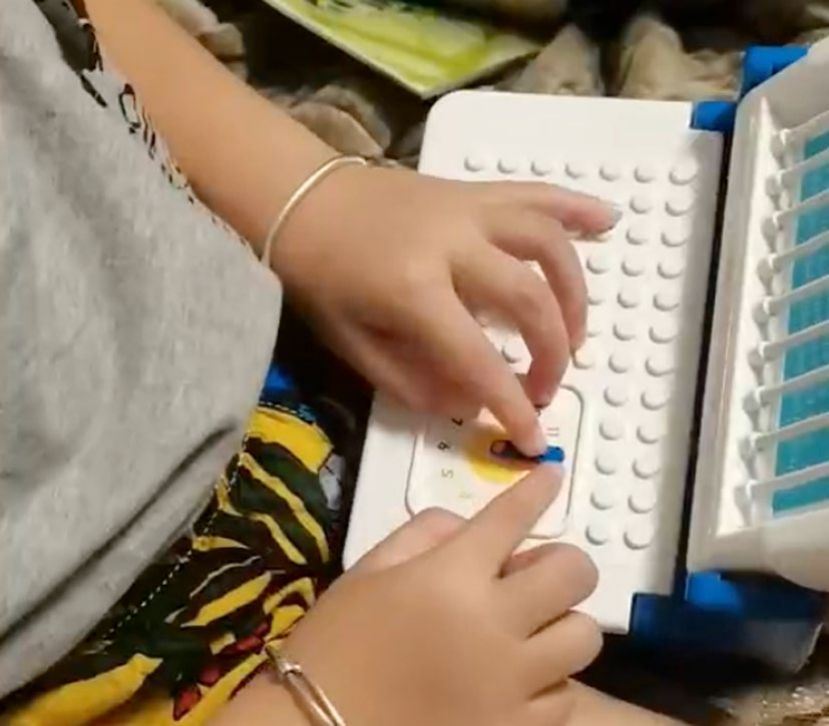
Child playing with toy. The screenshot from one of the diary video clips shows a child being attracted to the toy in his hand.

In *play* activities, *toys* were commonly used as *attractors* to capture the attention of children. For example, as observed in one diary clip where the son was obsessed with fiddling with the watch hands of a toy and reading the numbers aloud, the father repeated after the son’s words repeatedly (see Fig. 1) to keep the interaction going. The result that *toys* are commonly used as *attractors* in *play* activities may be explained that toys provide younger children with a high level of openness, which is essential for autistic children to create joint attention and maintain engagement. When *toys* were used as *attractors* in *play*, the initiative ratio for children (27.7%) was much higher than other objects. And it was also higher than when toys had other functions in play. However, the ratio of *Declarative* initiatives was extremely low, children were expressing their needs through requesting most of the time. Interaction clips also revealed that the children were particularly focused on the toys and hardly ever took their eyes off them. The responsiveness of child with *toys* in *play* activities (61.7%) was lower than the overall response rate compared to the other two types of objects, but for parents the number is the highest (97.2%). Children failed to include parents actively in the play with toys, they tended to focus on interaction with objects alone, which consists with general understanding of children with autism. We found that they initiated interactions only to get parents assisting them in playing with toys, not to play together with parents through toys. The difference is that children do not engage others in a cooperative and reciprocal way.

Except for being involved in *play* activities, *toys* were used as the *tool* to support *learning and practice*. *Toys* involved in this type were the most common ones for young children, for example those so-called Montessori toys^41^. This highlights the fact that these toys are not just playthings, but also serve as valuable tools for learning and development in various settings, such as schools and homes. When *toys* are involved under this condition, the designed playing method becomes the guideline, the measurable, and the goal of the practice, and the *toys* themselves become complete *tools*. In this case, less than 10% of interactions were initiated by children, but the proportion of children's *Confirming* responses was the highest among all types of objects in different activities (excluding accidental cases), with the proportion of declining responses being mainly in *Non-confirming* and *Neutral*. For parents, *Imperative* initiatives were dominant.

### Mood, evaluation, skills and goals, and importance

Compared to the *mood* of the family member, the *mood* of the child appeared to be related more closely with a wider variety of factors. When *daily necessities* were utilized in interactions with the child, a higher frequency of *non-confirming/non-compliant* responses were observed than *confirming* responses. It was noted that two interaction clips which predominantly featured *non-confirming* responses were both instances in which the child was labelled as feeling frustrated. This might suggest that interactions involving *daily necessities* may elicit more frustrated emotions in children. On the other hand, interactions involving toys were more likely to elicit feelings of excitement in children.

However, it is important to note that despite the child's *mood*, parents' evaluations of the interaction were not affected. Multiple interaction clips labelled as *frustrated* in terms of the *mood* of the child actually were labelled as *positive* in terms of parents’ evaluation, parents explained it in the diaries that “encourage the child to do his own things”, “let the child participate in the family activities and do something they can do”, other answers also suggested that parents value more about what the child did, the completion of the interaction and the importance of the interaction itself. For ‘*not important*’ interactions, parents almost rated them all as *neutral* regardless of the child's mood or performance. For interactions involving *toys*, the polarized evaluation results seemed to align with child’s *moods*, however for *daily necessities*, frustration did not necessarily relate with negative evaluations.

Our research uncovered a noteworthy insight regarding parents' daily interactions with their children. Child development goals were not found to be a primary focus of attention for parents during these interactions. Additionally, many of these interactions did not have a specific developmental purpose attached to them, despite professional guidelines which promote family interactions as a valuable opportunity for home intervention. While self-care was deemed an essential aspect in daily interactions, fine motor and social interaction were the most addressed skills and goals across various types of objects. Due to limited entries, the descriptors associated with these two skills and goals were tools, practice and learning, and positive evaluation.

### Influential factors of family interactions for young autistic children

Previous studies have explored various aspects of family interaction in the context of ASD, with a particular focus on parent–child interaction. For children, clear evidence had shown the effects of child’s gender, the level of severity of autism symptoms, their interaction style, and mood in dyadic interactions^1,20,36,37,42^. Potential factors including language ability^43^, adaptive behavior/functioning^44,45^, intelligence quotient and sensory preferences of the child^46,47^. There is a lack of research on the effect of these individual characteristics on the family interaction. For families, interaction strategy such as accommodation and reducing uncertainty were discovered to have strong effect on parent–child interaction, while reinforcement approaches were not related with child’s problem behaviors^17,48^. Poor psychological well-being of parents, commonly manifested as high levels of parenting stress and depressive symptoms, has been found to negatively affect the parent–child relationship^21,49^. Parent training has been shown to be effective in improving parent knowledge about ASD, enhancing parent–child interaction, and leading to positive child outcomes^50^. However, previous research has presented mixed findings on the relation between parents' education level and family quality of life, that includes family interaction^51,52^. Similarly, more evidence is needed to reveal the possible effects of other parental, demographic, and socio-economic factors (education attainment, family income occupation/employment level, cultural background) on the parent–child interaction^43,53,54^. Our own research has revealed that carers with higher education levels tended to have more positive interactions with their child, highlighting the need for more research to explore the influence of different levels of education among family members. Similarly, we discovered some signs of the different employment status influencing the parent–child interaction.

**Figure 2:**
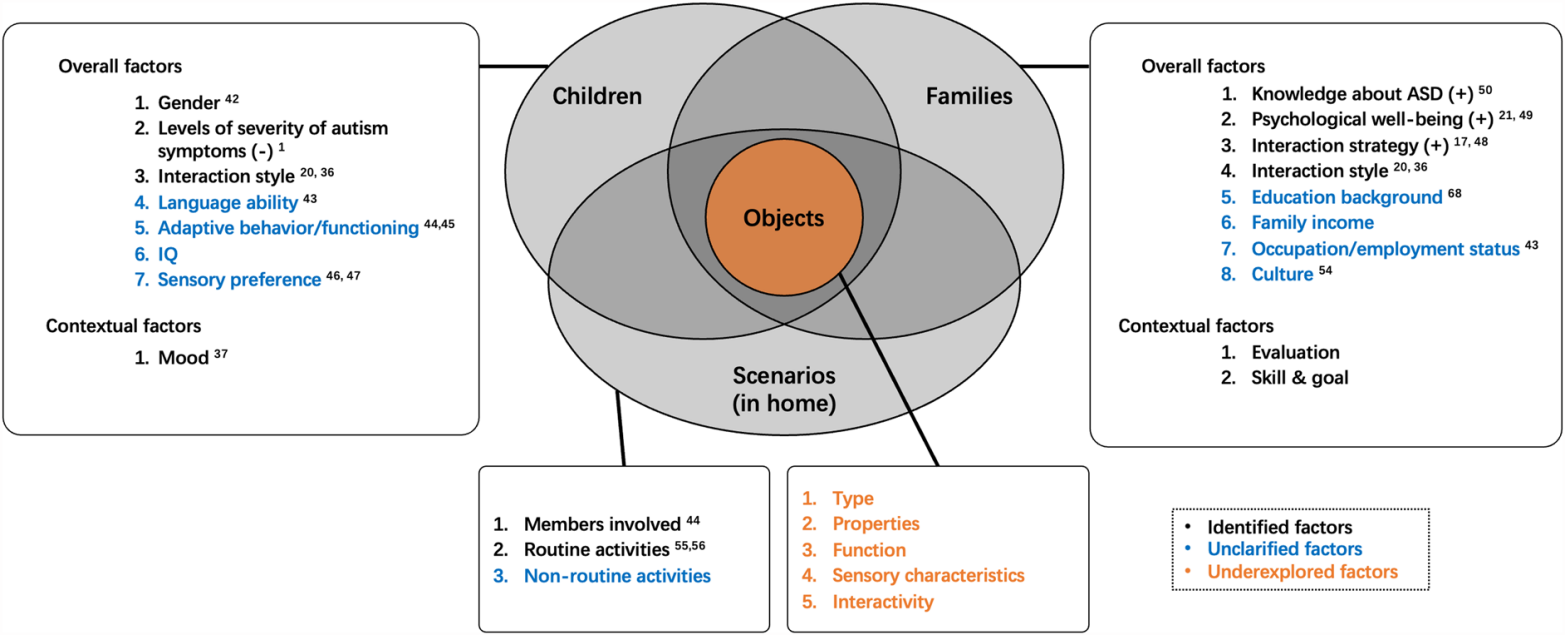
Factors in object-centered family interaction for young autistic children. The preliminary conceptual framework was developed based on a review of identified and unidentified factors of family interaction and for organizing existing knowledge and the findings of this study.

By integrating and organizing previous studies and analysis of this diary study. We propose a descriptive model of object-centered family interactions for young autistic children with identified influential factors and some unclarified but potential factors (Fig. 2). The model consists of four main elements with objects being the focus in the middle. For the child and family members, there are overall factors and situational factors. The overall factors serve as the foundation for understanding family interactions, while the contextual factors focus on the specific details of the interaction process. We discovered that the mood of the child, the evaluation of the interaction from the families and the skill and goal that involved during the interaction showed some signs of being potential contextual factors. Interactions integrated into routine activities had been explored in previous studies^55,56^, however, interactions happened in naturalistic contexts during non-routine activities still lack research.

The importance and value of objects in interaction for autistic individuals have been recognized in various studies^57,58^, however, the specific characteristics of different objects have not been explored in details. There is hardly any research on the design guideline or criteria for designing interactive objects for family interaction in autistic children. Based on related design studies^59^, together with the analysis and discoveries from interaction diaries, we propose several factors for exploring the effects of objects in this specific context.

When examining through interaction fragments, we found that factors such as activity type, object use, interaction style, and a child's moods can have a significant impact on the establishment, maintenance, and quality of the interaction. Further exploration is needed to examine all the colored descriptors/keywords in the Fig. 2.

## Discussion and Implications

The use of objects in this study partially aligned with our hypothesis on possible results, which *toys* accounted for the most majority of all interactions. However, the role it played in the family interaction is limited. On the contrary, *daily necessities*, although involved less in the collected interaction fragments, were used in different daily activities serving through multiple functions. *Toys* seem to be more limited by their special designed functions and purposes, and many existing studies had investigated the contribution of toy with autistic children, while we see unexplored potential in *daily necessities* in the field of HCI for developing future tangibles for enhancing family interactions. The Ignore rate of children’s response is very significant but forming joint attention is a key step for them during interaction^29^. Joint attention can either be achieved by attracting children’s attention or by following their focus and actively join in. Toys presented good examples in our study, and we believe other tangibles specially designed for autistic children could also be helpful, taking the advantages of the flexibility of HCI designs.

Recorded parents’ behaviors reflected that they were avoiding using instructions or command that are too complicated and difficult. While such guidance is important in establishing suitable boundaries for positive interaction and preventing frustration for the child, it fails to address whether the interaction is stimulating enough for the child to benefit from it. Study showed that scaffolding children during interactions to reach their potential development can lead to actual development in their skills and abilities^60^. We observed that some parents did offer some scaffoldings, but they were usually limited to basic practices like repetitive instructions and verbal encouragement. It is crucial to introduce families to more effective practices and strategies to promote higher quality parent–child interactions. We think existent techniques for parents could be supported with the involvement of tangible designs or HCI. Successful examples include tangible toys such as Polipo, designed with a cause-and-effect model for game play, and the Fisher-Price Smart Tablet, which could offer abundant educational materials^41^. However, still, there is a lack of exploration on other types of daily objects.

The significance of considering the holistic family system in the care of children with autism has been highlighted in previous research^61,62^, underscoring the crucial role of parents in the lives of autistic children. On one hand, studies have explored how parental involvement can contribute to the treatment of autistic children^63^, although there are still numerous aspects that warrant further investigation. On the other hand, the impact of caregiving for autistic children on parents should not be overlooked^64^. As mentioned earlier, poor psychological well-being of parents has been found to have a detrimental effect on parent–child relationships, which is often a consequence of the long-term, intensive, and challenging nature of caregiving. In fact, various factors such as emotional and behavioral problems (EBP) in children, parenting stress (PS), parental mental health problems (MHP), parental involvement (PRQ), intervention outcomes, and family functioning are interconnected and intertwined within the family system^61,65^. In our study, although we observed externalized behavior, we did not directly capture details pertaining to parental mental health and family involvement, which limited our ability to fully depict the interaction between parents and children. The intricate nature of these influencing factors and their interrelationships underscores the importance of interdisciplinary research in this field.

There are some limitations to our research. Firstly, the sample size of participants was relatively small, and we did not include any female subjects. It is worth noting that previous research has demonstrated significant differences between boys and girls with autism^42^. However, it is common for studies involving children with ASD to have small sample sizes. We observed that several studies focusing on parent–child interaction also had extremely small sample sizes^66^. Therefore, it is important that generalizing the conclusions drawn from this study should be done with caution. Further research is required to explore the implications of our findings across a broader demographic. Additionally, it is worth mentioning that fathers were essentially absent from this study, as is the case with most research on autism^67^. This may introduce a bias in the characteristics of parental interaction that were observed. Furthermore, in this study, the sample appeared to be skewed towards caregivers with limited formal education. However, the influence of parental educational level on parent–child interaction in statistical models needs to be further investigated^68^.

Diary studies, by their nature, rely on participants to proactively record information. As a result, the quality and quantity of data collected may have been influenced by the level of commitment from the parents. Additionally, despite the use of video observation to eliminate the presence of researchers, the knowledge of being recorded may have still affected the participants' behavior. To mitigate these potential biases, a more direct and long-term observation approach could be considered. Such an approach would help to reduce the influence of parents' perceptions, any conscious or subconscious covering up of the children's negative behavior, as well as practical challenges associated with unsettling the children.

## Conclusions

The establishment of interaction between ASD children and family members during activities posed certain challenges. Parents exhibited a lack of responsiveness and struggled to maintain their children's attention, resulting in a one-sided interaction pattern where parents predominantly initiated interactions and children responded by complying with their commands. The use of objects, particularly toys, proved to be beneficial in supporting these interactions by stimulating parents' enthusiasm and capturing children's attention. However, appropriate guidance was necessary to prevent excessive reliance on toys and ensure that children's social inclinations were not adversely affected. In addition, our research uncovered the untapped potential of employing everyday necessities as a promising avenue for future tangible design developments. We also observed a close relationship between children's mood and their performance in interactions, while parents' mood and their evaluation of the interaction appeared to be unaffected. Interestingly, parents placed greater value on the quality of the interaction process rather than solely focusing on the development of their children's abilities.

## Data Availability

The datasets generated and/or analyzed during the current study are not publicly available due to participants’ requirements but are available from the first author on reasonable request.
